# Effects of Smartphone-Based Stress Management on Improving Work Engagement Among Nurses in Vietnam: Secondary Analysis of a Three-Arm Randomized Controlled Trial

**DOI:** 10.2196/20445

**Published:** 2021-02-23

**Authors:** Natsu Sasaki, Kotaro Imamura, Thuy Thi Thu Tran, Huong Thanh Nguyen, Kazuto Kuribayashi, Asuka Sakuraya, Thu Minh Bui, Quynh Thuy Nguyen, Nga Thi Nguyen, Giang Thi Huong Nguyen, Melvyn Weibin Zhang, Harry Minas, Yuki Sekiya, Kazuhiro Watanabe, Akizumi Tsutsumi, Akihito Shimazu, Norito Kawakami

**Affiliations:** 1 Department of Mental Health Graduate School of Medicine The University of Tokyo Tokyo Japan; 2 Department of Occupational Health and Safety Faculty of Environmental and Occupational Health Hanoi University of Public Health Hanoi Vietnam; 3 Faculty of Social Sciences—Behavior and Health Education Hanoi University of Public Health Hanoi Vietnam; 4 Department of Public Health Tokyo Women's Medical University Tokyo Japan; 5 Nursing Office Bach Mai Hospital Hanoi Vietnam; 6 National Addiction Management Service Institute of Mental Health Singapore Singapore; 7 Melbourne School of Population and Global Health The University of Melbourne Melbourne Australia; 8 Department of Public Health Kitasato University School of Medicine Sagamihara Japan; 9 Faculty of Policy Management Keio University Kanagawa Japan

**Keywords:** stress management, mental health, occupational health, digital health, workplace, LMICs, South-East Asia, health care professionals

## Abstract

**Background:**

Work engagement is important for employee well-being and work performance. However, no intervention study has investigated the effect of an eMental Health intervention on work engagement among workers in low- and middle-income countries (LMICs).

**Objective:**

The aim of the study was to examine the effects of a newly developed smartphone-based stress management program (ABC Stress Management) on improving work engagement among hospital nurses in Vietnam, an LMIC.

**Methods:**

Full-time registered nurses (n=949) were randomly assigned to one of 2 intervention groups or a control group. The intervention groups were a 6-week, 6-lesson program offering basic cognitive behavioral therapy (CBT-based stress management skills), provided in either free-choice (program A) or fixed order (program B). Work engagement was assessed at baseline and 3-month and 7-month follow-ups in each of the 3 groups.

**Results:**

The scores of work engagement in both intervention groups improved from baseline to 3-month follow-up, and then decreased at the 7-month follow-up, while the score steadily increased from baseline to 7-month follow-up in the control group. Program B showed a significant intervention effect on improving work engagement at the 3-month follow-up (*P*=.049) with a small effect size (Cohen *d*= 0.16; 95% CI 0.001 to 0.43]). Program A showed nonsignificant trend (*d*=0.13; 95% CI –0.014 to 0.41; *P*=.07) toward improved engagement at 3 months. Neither program achieved effectiveness at the 7-month follow-up.

**Conclusions:**

The study demonstrated that a fixed order (program B) delivery of a smartphone-based stress management program was effective in improving work engagement in nurses in Vietnam. However, the effect was small and only temporary. Further improvement of this program is required to achieve a greater effect size and more sustained, longer lasting impact on work engagement.

**Trial Registration:**

University Hospital Medical Information Network Clinical Trials Registry UMIN000033139; tinyurl.com/55gxo253

**International Registered Report Identifier (IRRID):**

RR2-10.1136/bmjopen-2018-025138

## Introduction

Work engagement is a popular topic in occupational mental health, given its positive impact on employee well-being and work performance [1,2]. Increasing and sustaining work engagement is therefore a prime concern of many organizations. Work engagement is one of the positive mental health outcomes resulting from a positive, fulfilling, work-related state of mind. It has 3 dimensions: vigor, dedication, and absorption [3]. Engaged workers who are connected to the work environment and its activities have sufficient energy to meet the demands of their job, perform well, and have low intention to leave [4]. Many individual and group-based [1,5] intervention programs have been developed to improve work engagement, including personal resource building (eg, resilience training), job resource building (eg, increased social support and feedback), leadership training (eg, skills improvement for managers), and health promotion (eg, stress management). A promising option would be an internet-delivered eHealth intervention that is feasible, low-cost, effective, and accessible [6-8]. An earlier randomized trial including an internet-based cognitive behavioral therapy (iCBT) intervention in the form of a self-care program showed a small effect size (d=0.16) and improved work engagement among workers [9]. Another web-based stress management literacy intervention that included CBT components also improved work engagement among participants with lower baselines of work engagement [10]. An iCBT intervention may be an affordable, sustainable, and effective approach to improving work engagement.

Smartphone ownership has been rapidly increasing in low- and middle-income countries (LMICs) [11], and they seem to be ready for eHealth implementation. In Vietnam in 2017, for instance, the percentage of people using smartphones in rural and urban areas was 68% and 84%, respectively [12]. In LMICs, work engagement has also been associated with better health, greater life satisfaction, and improved work performance [13-15]. An iCBT intervention may increase work engagement in low-resource settings such as LMICs. However, to date, no intervention study has investigated the effect of an iCBT or any other eMental Health intervention on work engagement among workers in LMICs.

Engagement in their work holds an important role in nurses’ health, well-being [14,16], work performance; the quality of health care services they provide [17-19]; and turnover intentions [20-22]. In addition, nursing is a stressful profession with high work-related stress and burnout in both high-income countries and LMICs [23]. In Vietnam, job demands have increased because of the shortage of nurses [24-26] and the rapidly aging society [27-29]. Work engagement can help nurses manage work-related stress [17,30]. Two previous studies have investigated the effect of web-based interventions on improving nurses’ work engagement in Germany, a high-income country [31,32]. The findings were inconsistent: one showed significant effectiveness [32] and the other did not [31]. It is important to know if an iCBT intervention will indeed increase work engagement among nurses in LMICs.

The purpose of this study was to determine whether a newly developed smartphone-based stress management program improved work engagement among hospital nurses in Vietnam. We analyzed data collected in a randomized controlled trial (RCT) that included work engagement as a secondary outcome [33].

## Methods

### Trial Design

This was a 3-armed RCT (allocation ratio: 1:1:1) examining improvement of depressive and anxiety symptoms as primary outcomes and, as stated, work engagement as a secondary outcome. The results of two internet-based CBT stress management programs (free-choice sequence and fixed sequential order) were compared with that of a control group at 3-month and 7-month follow-ups among hospital nurses in Vietnam [33]. The research ethics review board of the Graduate School of Medicine/Faculty of Medicine, University of Tokyo (No. 11991) and the ethical review board for Biomedical Research of Hanoi University of Public Health (No. 346/2018/YTCC-HD3) approved the study procedure. The study protocol was registered at the University Hospital Medical Information Network Clinical Trials Registry [UMIN000033139]. The trial protocol is published elsewhere [33]. This manuscript conforms to the Consolidated Standards of Reporting Trials (CONSORT) guidelines [34,35].

### Participants

All participants (n=1256) were recruited from a large public tertiary hospital at national level in Hanoi, Vietnam in 2018. We distributed written information about the study, consent form, baseline questionnaires, and a numbered envelope to return the completed questionnaires anonymously. The inclusion criteria were being employed full time as a registered nurse and having internet access via a mobile device such as a smartphone. The exclusion criteria were (1) plans to change or leave the job in the next 7 months, (2) being an assistant nurse and helper, (3) being temporary or part-time employed, (4) having taken leave for 15 or more days for a physical or mental condition in the past 3 months, and (5) undergoing treatment for a mental health problem from a mental health professional. However, exclusion criteria 4 and 5 were withdrawn before the start of the baseline survey (see Changes to the Protocol in Methods).

### Intervention Programs

Two smartphone-based stress management programs (program A and program B) were developed in the ABC Stress Management app. Program A was a free-choice multimodule program, in which participants could select a module to complete each week in any order. Program B was a fixed-sequential order multimodule program, in which participants were required to complete one module per week in a fixed order. The contents of program A were based on a previous online stress management program to reduce the distress of office workers in Japan [36]. Program B included CBT-based stress management skills adapted from a previous iCBT program that reduced depressive symptoms in Japanese office workers [37]. Each program contained 6 modules. It took about 15 minutes to complete one module. We developed the programs based on discussions with Vietnamese nurses to consider the cultures and specific stressors that they could have at work. Several meetings were held to allow 30 head nurses to share their stressful experiences at work and their reflections on the draft program content; these head nurses were also invited to participate in reviewing the programs, and the programs were revised based on their feedback. Full details of these programs can be found in the published study protocol paper [33]. The mp4 file of the programs can be found in Multimedia Appendix 1.

### Intervention Groups (Programs A and B)

Participants in the intervention groups were required to complete program A or B within 10 weeks after the baseline survey. Participants began the program after signing in with their ID and password. The clinical research coordinator sent weekly reminder messages to people who had not completed a module on time. An informal group chat (via social media apps such as Viber, Zalo, Facebook Messenger) with researchers and hospital head nurses was used to deliver intensive technical support for participants to complete the program. Before the start of the intervention, researchers helped participants download the app and complete an introductory module that provided a general explanation of how to use the app.

### Control Group

Participants in the control group did not receive any intervention during the 7-month intervention period. However, they were free to use any other mental health service as usual treatment. Intervention programs were provided for the control group after the 7-month follow-up.

### Outcome Measurement

All outcomes were assessed at baseline and, 3-month (the end of the intervention period), and 7-month follow-ups with a paper-based self-administered survey questionnaire. Participants were allowed 10 weeks to complete the program (6 weeks for the modules and 4 weeks to review any modules they desired). Follow-up at 3 months was timed to assess immediate effects of the interventions. For administrative reasons, we conducted a follow-up at 7 months, instead of a usual 6-month follow-up, to evaluate longer term effects.

#### Work Engagement

The short form of the Utrecht Work Engagement Scale–9 item (UWES-9) was used to assess work engagement [3]. The UWES-9 consists of 3 subscales (vigor, dedication, and absorption) that contain 3 items each. The UWES-9 uses a self-report 7-point rating scale (0= never; 6= every day). The mean scores of the 3 UWES subscales and the total score are computed by adding the scores and dividing the sum by the number of items in each subscale. Hence, the UWES’s 3 subscale scores and a total score range from 0 to 6. The Vietnamese version of UWES-9 has been validated elsewhere [38]. The Cronbach alpha coefficients of the UWES-9 and vigor, absorption, and dedication subscales were .93, .86, .77, and .90, respectively. Confirmatory factor analyses indicated that the 3-factor structure was acceptable.

#### Demographic Variables

Demographic and occupational variables were assessed using a questionnaire and included gender (male or female), age, education (vocational school, college, university undergraduate, or postgraduate), marital status (single, married or divorced/widowed), and employment contract (fixed-time contract for less than one 1 year, fixed‐time contract for more than 1 year, unlimited time contract, permanent contract, or other). Age was calculated based on the year of birth.

### Sample Size Calculation

The sample size in the study was calculated for the primary outcome (ie, depressive symptoms assessed by the Depression, Anxiety, and Stress Scale–21-item [DASS-21]) when the effect size was set to 0.25. A post hoc test power (1-beta) for work engagement was calculated as 0.52 at the 3-month follow-up and 0.10 at the 7-month follow-up, assuming that the alpha was less than 0.05 (2-tailed) and the number of respondents in each group who were included in the analyses was 317, using the G*Power 3.1 program (Heinrich-Heine-Universität Düsseldorf) [39,40].

### Randomization

Eligible participants were randomly assigned to one of the 3 trial arms (2 intervention groups and a control group). Stratified permuted-block randomization was conducted. The block sizes were fixed at 3. Participants were stratified according to the baseline depression subscale score of DASS-21 into 2 strata (≥10 or <10) [41]. A stratified permuted block random table was generated by an independent biostatistician. Enrollment was conducted by a clinical research coordinator (TTTT), and the assignment was conducted by an independent research assistant. The stratified permuted-block random table was password-protected and blinded to the researchers. Only the research assistant could access it during random allocation.

### Statistical Analyses

For the main pooled analysis, a mixed model for repeated measures conditional growth model analysis with an unstructured covariance matrix was conducted using a group (intervention and control) × time (baseline and 3-month and 7-month follow-ups) interaction as an indicator of the intervention effect. The 2 intervention effects (program A vs control and program B vs control) were simultaneously tested in the model. For sensitivity analysis, a similar mixed model for repeated measures, but using the analysis of variance model with an unstructured covariance matrix, was conducted.

At the baseline survey, if the number of missing items was less than half of the number of total items, the missing values were imputed, using values calculated according to the following equation: (mean value × total number of items) / number of missing items. Otherwise, if the number of missing items was more than half of the total items, the case was not imputed and treated as missing. Missing values at follow-up surveys were imputed by applying the maximum likelihood estimation. An intention-to-treat principle was applied.

The effect size was estimated in 2 ways. First, we estimated a regression coefficient for a group (each of the 2 intervention groups vs the control group) × time (baseline and 2 follow-ups) interaction, which was converted to an effect size by dividing by a pooled standard deviation at baseline and follow-ups. Second, we calculated Cohen *d* among completers at baseline for each follow-up.

Statistical significance was defined as *P*<.05. All statistical analyses were performed using SPSS Statistics 26.0, Japanese version (IBM Corp).

### Changes to the Protocol

Before starting the study, 2 of the exclusion criteria (no. 4 and no. 5, described above) were removed because these restrictions would be expected to reduce the participation rate, and because reducing the eligibility restrictions when selecting participants is desirable for the purpose of ascertaining the effect of the programs in the real setting as a pragmatic trial [42].

## Results

### Characteristics of Participants

**Figure 1:**
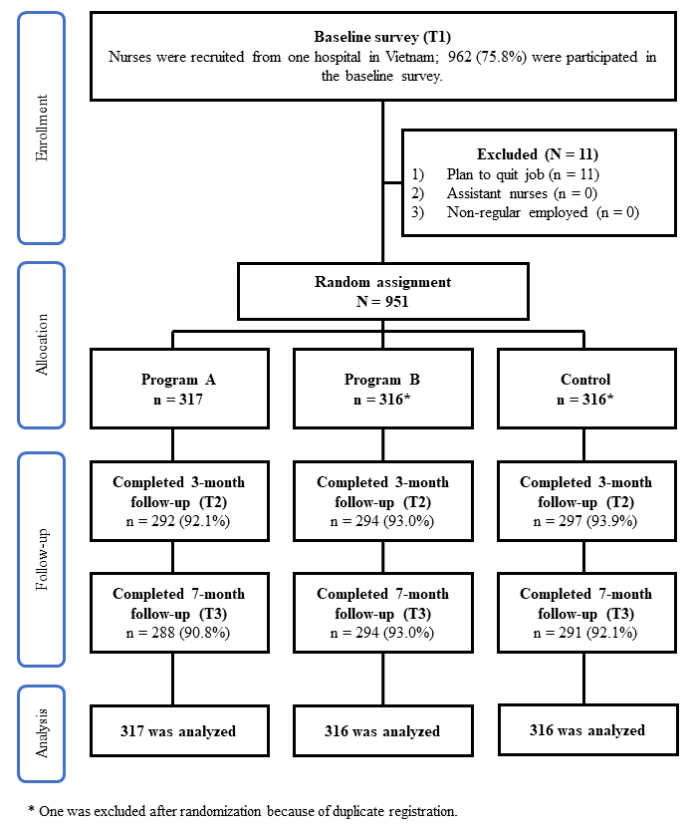
Participant flowchart.

The participant flowchart is shown in Figure 1. In total, 75.81% (962/1269) of nurses participated in the baseline survey (September 2018). After 11 were excluded, 951 met the eligibility criteria. Finally, the 951 participants were randomly allocated with 317 in each group (2 interventions and one control group). After the random assignment, one participant in the intervention group (program B) and one in the control group were excluded because of duplicate registration. The follow-up rates were 92.11% to 93.04% (3 months, January 2019) and 90.85% to 93.04% (7 months, May 2019). The baseline characteristics of participants are shown in Table 1. Most (806/949, 84.9%) of the participants were female, nearly half (443/949, 46.7%) had graduated from vocational school, and the majority (793/949, 83.6%) were married. Employment contracts of most nurses were permanent (505/949, 53.2%). The average age was 33.1 years (range 22 to 58).

**Table 1:** Demographic characteristics of study participants in the intervention and control groups of hospital nurses in Vietnam (n=949).

Characteristics		Program A (n=317)	Program B (n=316)	Control (n=316)
**Gender, n (%)**				
	Male	39 (12.3)	56 (17.7)	48 (15.2)
	Female	278 (87.7)	260 (82.3)	268 (84.4)
Age in years, mean (SD)		33.7 (7.3)	32.8 (6.6)	32.8 (6.4)
**Education level, n (%)**				
	Vocational school	151 (47.6)	142 (44.9)	150 (47.5)
	College	39 (12.3)	50 (15.8)	48 (15.2)
	University undergraduate	118 (37.2)	119 (37.7)	111 (35.1)
	Postgraduate	6 (1.9)	5 (1.6)	6 (1.9)
	Unknown	3 (0.9)	0 (0)	1 (0.3)
**Marital status, n (%)**				
	Single	40 (12.6)	44 (13.9)	53 (16.8)
	Married	270 (85.2)	267 (84.5)	256 (81.0)
	Divorced/widowed	5 (1.6)	5 (1.6)	6 (1.9)
	Unknown	2 (0.6)	0 (0)	1 (0.3)
**Employment contract, n (%)**				
	Fixed-time, ≤1 year	64 (20.2)	79 (25.0)	77 (24.4)
	Fixed-time, >1 year	10 (3.2)	8 (2.5)	12 (3.8)
	Unlimited time	62 (19.6)	69 (21.8)	63 (19.9)
	Permanent	181 (57.1)	160 (50.6)	164 (32.5)

### Effect of the Intervention Programs on Work Engagement

The average scores of work engagement and subscales at each time are shown in Table 2. The scores of work engagement in both intervention groups improved from baseline to 3-month follow-up but slightly decreased by the 7-month follow-up. The score in the control group increased at 3-month and 7-month follow-ups. Table 3 shows the estimated effect of both interventions on improving work engagement. Program B showed a significant estimated effect for improving work engagement at 3 months (*t*=1.97, *P*=.049, *d*=0.16; 95% CI 0.001 to 0.43). On the other hand, program A showed a nonsignificant trend (*t*=1.82, *P*=.069, *d*=0.13; 95% CI –0.014 to 0.41) toward improved work engagement at 3 months. At 7 months, neither program showed a significant effect (program A: *t*=0.02, *P*=.98, *d*=–0.004; 95% CI –0.17 to 0.16; program B: *t*=0.65, *P*=.52, *d*=0.05; 95% CI –0.11 to 0.21).

**Table 2:** The mean scores of the Utrecht Work Engagement Scale–9 item among intervention and control groups from baseline to 3-month and 7-month follow-ups and effect size (Cohen d).

Survey	Program A (n=317)		Program B (n=316)		Control (n=316)		Program A vs control	Program B vs control
UWES-9^a^ (range 0-6)	n	mean (SD)	n	mean (SD)	n	mean (SD)	Cohen *d*^b^ (95% CI)	Cohen *d*^b^ (95% CI)
Baseline	317	4.3 (1.2)	316	4.3 (1.3)	316	4.3 (1.3)	—	—
3 months	292	4.6 (1.2)	294	4.7 (1.1)	297	4.4 (1.3)	0.13 (–0.03 to 0.29)	0.16 (0.002 to 0.32)^c^
7 months	288	4.5 (1.3)	294	4.6 (1.1)	290	4.5 (1.3)	–0.004 (–0.17 to 0.16)	0.05 (–0.11 to 0.21)

^a^UWES: Utrecht Work Engagement Scale–9 item.

^b^Effect sizes were calculated among completers at baseline for each follow-up.

^c^*P*=.049.

**Table 3:** The estimates of fixed effects and effect size at 3-month and 7-month follow-ups for work engagement in both intervention groups (programs A and B).

Time points		Estimates of fixed effects					Estimated effect size
		Effect size	SE	95% CI	*t* score	*P* value	
**Program A**							
	3-month^a^	0.20	0.11	–0.02 to 0.41	1.82	.07	0.14
	7-month^a^	0.003	0.12	–0.23 to 0.23	0.02	.98	0.002
	Pooled^b^	0.005	0.06	–0.11 to 0.12	0.08	.94	—
**Program B**							
	3-month^a^	0.21	0.11	0.001 to 0.43	1.97	.049	0.16
	7-month^a^	0.08	0.12	–0.15 to 0.30	0.65	.52	0.05
	Pooled^b^	0.04	0.06	–0.08 to 0.16	0.67	0.5	—

^a^Mixed-model for repeated measures analysis of variance model analyses was conducted.

^b^Mixed-model for repeated measures conditional growth model analyses was conducted.

## Discussion

### Principal Findings

The 3-arm RCT examined the effects of a newly developed smartphone-based stress management program to improve work engagement as the secondary outcome at 3-month and 7- month follow-ups among hospital nurses in Vietnam. Program B, a 6-module CBT program with fixed sequential order, showed a significant small intervention effect on work engagement at the 3-month follow-up (*d*=0.16, *P*=.049). Program A did not show a significant effect at the 3-month follow-up. Neither program achieved effectiveness at the 7-month follow-up. A smartphone-based stress management program with fixed sequential order may be effective in improving work engagement in a population of nurses in an LMIC, Vietnam.

Scores on the UWES-9 increased (indicating improved work engagement) from baseline to seven-month follow-up in the control group as well. A possible reason is potential contamination by information provided about the intervention programs from intervention groups to the control group [38]. Control group participants may have received information on stress management from their colleagues who were in the intervention groups. Other reasons may include a seasonal workload change or organizational reforms in the target hospital.

Program B significantly improved work engagement at the 3-month follow-up among nurses in the intervention groups compared with nurses in the control group. This result was consistent with a previous RCT on work engagement among information technology workers in Japan [9], which showed that a similar web-based, traditional CBT intervention with fixed-order modules achieved a significant but small intervention effect. This study expands the evidence on the effectiveness of iCBT on work engagement from workers in a high-income country [9] to hospital nurses in Vietnam, a LMIC.

In terms of the feasibility and reach of a smartphone-based intervention, more than 80% of nurses in the target hospital owned smartphones. The content of the programs was appropriate and relevant since the case story presented was based on interviews with nurses in Vietnam about managing their stress on the job. After making an outline, the content was reviewed and confirmed by nurses. These factors may facilitate the use of program B and support its ability to improve work engagement. However, the effectiveness of an iCBT program may depend on the culture and internet or smartphone literacy in a target country. Moreover, nurses are professionals. Their job requires an advanced college or university education. Additional research is needed to expand these findings to other LMICs and occupations.

Nurses who used program B were required to learn the cognitive behavioral model in an early module before continuing to later modules. Studying cognitive restructuring skills may decrease negative emotions [43], enhance positive cognitions concerning work-related challenges, and increase work engagement. It was reported that emotion-regulating cognitive styles, such as positive reinterpretation, focus on plans, acceptance, positive focus, and putting into perspective, are associated with work engagement [44]. Thus, cognitive styles changed by the cognitive restructuring skill may directly improve work engagement. It is also possible that adaptive coping skills learned from program B may increase personal resources (eg, self-efficacy [45]) and psychosocial job resources (eg, workplace social support) by helping employees achieve work goals and stimulate personal learning and development [46,47]. The mechanism by which an iCBT program improves work engagement needs to be clarified in future research.

There was no significant effect of program B on work engagement at 7 months. A previous review of eHealth interventions indicated that the long-term benefits of an eHealth program are still unknown due to small effect size [48]. In this study, it is plausible that participants forgot what they had learned because of its lack of intensity and repeatability. To improve long-term effectiveness, refresher sessions may be needed after the intervention period. Another reason might be the small sample size for proving the effectiveness on work engagement. A post hoc power analysis revealed that a sample of 615 in a group might be needed to detect the effect size (*d*=0.16) for 1-beta over .80. Further study should be conducted to examine long-term effectiveness for hospital nurses.

Program A shows only nonsignificant effect on work engagement at 3-month (*d*=0.16, *P*=.07) and 7-month follow-up. One study in a Western country reported that tailored (ie, partially personalized) options provided a better outcome of eHealth programs because free choice was culturally preferred [49]. Our result was inconsistent with that study. Perhaps the more collectivist and hierarchically oriented cultures in Asian countries are associated with people feeling more comfortable doing as others do and following instructions [50]. The free-choice style of program A might be a psychological burden for participants in Vietnam since they had to think about which module to read each week. Another reason was the difference in content between the 2 programs: program A did not intensively repeat cognitive restructuring skills or follow a sequential learning order of standardized CBT due to free-choice operation. A process evaluation showed similar completion rates for both programs (83.3% for program A and 86.1% for program B). A significant difference of process evaluation such as satisfaction and usefulness was not found (available upon request). The free-choice order program without intensive restructuring skills might not have a significant effect on Vietnamese nurses.

### Strengths and Limitations

The strengths of this RCT are the type of program delivery and high completion rate. The iCBT programs used in this study is fully automated and self-guided. Considering the small effect size, a self-guided program still has the merits of greater accessibility and lower cost than interactive or specialist-guided programs. This study had a high completion rate. For evaluating the cost-effectiveness and sustainability of such a program, it will be necessary to observe completion rates in practical settings without intensive reminders. Despite the small effect size, program B appears to be useful in improving nurses’ work engagement, with expected benefits for their health, quality of their services, and patient clinical outcomes. Successful dissemination and implementation of internet-delivered stress management for nurses will require collaboration between policymakers and stakeholders.

The study has several limitations. First, participants were recruited from a large and prestigious national general hospital in Hanoi, and were limited to full-time nurses with a personal smartphone. Therefore, the generalizability of these findings to the wider nursing population is limited. Second, this study did not adopt a stratified randomized method by using work engagement scores. Therefore, we did not conduct a subgroup analysis. Further studies on improving work engagement by stratified randomization might achieve a larger effect size in the low work engagement population. Third, all outcomes in this study were measured by self-report, which might be affected by participant perceptions or institutional factors. Future studies should consider the use of additional objective outcome measures such as supervisor ratings of work performance. Fourth, the possibility of contamination of information to the control group may have reduced differences between intervention and control groups, resulting in possible underestimation of intervention effectiveness not fully controlled in this study. Fifth, besides the intervention programs, an informal group chat (via Viber, Zalo, Facebook Messenger) led by researchers and hospital head nurses used to increase the participation rate may also have contributed to improvement of work engagement in the intervention groups. Sixth, there may have been social pressure or frustrations caused by frequent reminding to study the programs, reducing the effect of the program on work engagement. Seventh, the programs were designed mainly to target smartphone users, but they can be used on a PC or tablet. Nurses who did not have smartphones or internet access were excluded from access to the programs. In a future trial, in addition to the smartphone-based program, the same content in the programs should also be provided via other delivery modes such as computer, tablet, or booklet. Finally, it is not known whether a similar effect of this program would be observed outside of the nursing profession, in Vietnam or in other LMICs. Future studies should explore the generalizability of our findings to occupations other than nurses and in other LMICs.

### Conclusion

The study demonstrated that a smartphone-based stress management program with fixed order significantly improved work engagement in a working population of nurses in an LMIC, Vietnam. However, the effect of the intervention was small and temporary. Further improvement of this program is necessary to achieve a greater effect size and more sustained impact on work engagement. However, fully automated and self-guided programs with great accessibility and minimal cost can be well suited for LMICs. The generalizability of these findings to occupations other than nursing and in other LMICs should be investigated in future studies.

## Appendix

Multimedia Appendix 1 The ABC stress management program.

Multimedia Appendix 2 CONSORT-EHEALTH V1.6.1 checklist.

## Abbreviations

CONSORT: Consolidated Standards of Reporting Trials
DASS-21: Depression, Anxiety, and Stress Scale–21-item
iCBT: internet-based cognitive behavioral therapy
LMICs: low- and middle-income countries
RCT: randomized controlled trial
UMIN: University Hospital Medical Information Network Clinical Trials Registry
UWES-9: Utrecht Work Engagement Scale–9 item
