# Supplementary material for: Effects of Smartphone-Based Stress Management on Improving Work Engagement Among Nurses in Vietnam: Secondary Analysis of a Three-Arm Randomized Controlled Trial
Source: J Med Internet Res. 2021 Feb 23;23(2):e20445. doi: 10.2196/20445 (PMC7943341; doi:10.2196/20445)
Supplement: Multimedia Appendix 2 [file jmir_v23i2e20445_app2.pdf]

CONSORT-EHEALTH Checklist V1.6.2 Report	Manuscript Number	20445

(based on CONSORT-EHEALTH V1.6), available at [http://tinyurl.com/consort-ehealth-v1-6].

Date completed

8/31/2020 20:14:32

by

Norito Kawakami

Effects of smartphone-based stress management on improving work engagement among nurses in Vietnam: a secondary analysis of a three-arm randomized controlled trial

TITLE

1a-i) Identify the mode of delivery in the title

Effects of "smartphone-based stress management" on improving work engagement among nurses in Vietnam: a secondary analysis of a three-arm randomized controlled trial

1a-ii) Non-web-based components or important co-interventions in title

1a-iii) Primary condition or target group in the title

Effects of smartphone-based stress management on improving work engagement "among nurses in Vietnam": a secondary analysis of a three-arm randomized controlled trial

ABSTRACT

1b-i) Key features/functionalities/components of the intervention and comparator in the METHODS section of the ABSTRACT

"The intervention groups were a 6-week, 6-lesson program offering basic cognitive behavioral therapy (CBT-based stress management skills), provided in either free-choice (Program A) or fixed order (Program B)."

1b-ii) Level of human involvement in the METHODS section of the ABSTRACT

1b-iii) Open vs. closed, web-based (self-assessment) vs. face-to-face assessments in the METHODS section of the ABSTRACT

1b-iv) RESULTS section in abstract must contain use data

1b-v) CONCLUSIONS/DISCUSSION in abstract for negative trials

INTRODUCTION

2a-i) Problem and the type of system/solution

"However, to date, no intervention study has investigated the effect of an iCBT or any other eMental Health intervention on work engagement among workers in LMICs."

2a-ii) Scientific background, rationale: What is known about the (type of) system

"A promising option would be an Internet-delivered digital health (eHealth) intervention that is feasible, low-cost, effective, and accessible [6-8]. "

Does your paper address CONSORT subitem 2b?

"The purpose of this study was to examine the effects of a newly developed smartphone-based stress management program on improving work engagement among hospital nurses in Vietnam. "

METHODS

3a) CONSORT: Description of trial design (such as parallel, factorial) including allocation ratio

Data for this study were collected as secondary outcomes of "a three-armed randomized controlled trial (RCT) (allocation ratio: 1:1:1) " examining improvement of depressive and anxiety symptoms as primary outcomes.

3b) CONSORT: Important changes to methods after trial commencement (such as eligibility criteria), with reasons

Details are described in the "Changes to the protocol" section. However, we did not change the content.

3b-i) Bug fixes, Downtimes, Content Changes

4a) CONSORT: Eligibility criteria for participants

"All participants (N = 1,256) were recruited from a large public tertiary hospital in Hanoi, Vietnam. Written information about the study, a consent form, the baseline questionnaires and a numbered envelope to return the completed questionnaires anonymously were distributed. The inclusion criteria were: (1) employed full time as a registered nurse, and (2) Internet access via a mobile device such as a smartphone. The exclusion criteria were: (1) plan to change or leave the job in the next seven months, (2) being assistant nurses and helpers, (3) being non-regular or part-time employed, (4) having taken leave for 15 or more days for a physical or mental condition in the past three months, and (5) undergoing treatment for a mental health problem from a mental health professional. However, exclusion criteria 4 and 5 were withdrawn before the start of the baseline survey (see details in “Changes to the protocol” in Methods)."

4a-i) Computer / Internet literacy

4a-ii) Open vs. closed, web-based vs. face-to-face assessments:

"All participants (N = 1,256) were recruited from a large public tertiary hospital in Hanoi, Vietnam. " "Participants in the intervention groups were required to complete Program A or B within 10 weeks of the baseline survey." "Before the start of the intervention, researchers helped participants to download the app and complete an introductory module that provided a general explanation of how to use the app."

4a-iii) Information giving during recruitment

4b) CONSORT: Settings and locations where the data were collected

"All participants (N = 1,256) were recruited from a large public tertiary hospital in Hanoi, Vietnam."

4b-i) Report if outcomes were (self-)assessed through online questionnaires

All outcomes were assessed at baseline, 3-month (the end of the intervention period), and 7-month follow-ups with "a paper-based self-administered survey questionnaire."

4b-ii) Report how institutional affiliations are displayed

5) CONSORT: Describe the interventions for each group with sufficient details to allow replication, including how and when they were actually administered

5-i) Mention names, credential, affiliations of the developers, sponsors, and owners

5-ii) Describe the history/development process

5-iii) Revisions and updating

5-iv) Quality assurance methods

5-v) Ensure replicability by publishing the source code, and/or providing screenshots/screen-capture video, and/or providing flowcharts of the algorithms used

5-vi) Digital preservation

5-vii) Access

"Before the start of the intervention, researchers helped participants to download the app and complete an introductory module that provided a general explanation of how to use the app."

5-viii) Mode of delivery, features/functionalities/components of the intervention and comparator, and the theoretical framework

"Two smartphone-based stress management programs (Program A and Program B) were developed in “ABC stress management ” apps. Program A was a free-choice multi-module program, in which participants could select modules to complete in any order. Program B was a fixed-sequential order multi-module program, in which participants were required to complete one module per week in a fixed order. The contents of Program A were based on a previous online stress management program to reduce the distress of office workers in Japan [37]. Program B included CBT-based stress management skills, adapted from a previous iCBT program that reduced depressive symptoms in Japanese office workers [38]. Both programs contained six modules. It took about 15 minutes to complete one module. We developed the programs based on discussions with Vietnamese nurses to consider the cultures and specific programs that they could have at work. Several meetings were held with 50 head nurses to hear about their stressful situations at work and their reflections on the draft program content. These head nurses were also invited to review the programs and give their feedback and suggest revisions of the programs. Full details of these programs can be found in the published study protocol paper [34]. The movie image (mp4 file) of the programs can be found in Multimedia Appendix 1. "		
**5-ix) Describe use parameters**		
**5-x) Clarify the level of human involvement**		
**5-xi) Report any prompts/reminders used**		
"The clinical research coordinator (CRC) sent weekly reminder messages to people who had not completed a module on time. An informal group chat (via Viber, Zalo, Facebook Messenger) with researchers and hospital head nurses was used to deliver intensive support for participants to complete the program."		
**5-xii) Describe any co-interventions (incl. training/support)**		
N/A		
**6a) CONSORT: Completely defined pre-specified primary and secondary outcome measures, including how and when they were assessed**		
"The scores of work engagement in both intervention groups improved from baseline to 3-month follow-up but slightly decreased by the 7-month follow-up. The score in the control group kept increasing from baseline to 7-months."		
**6a-i) Online questionnaires: describe if they were validated for online use and apply CHERRIES items to describe how the questionnaires were designed/deployed**		
**6a-ii) Describe whether and how “use” (including intensity of use/dosage) was defined/measured/monitored**		
**6a-iii) Describe whether, how, and when qualitative feedback from participants was obtained**		
**6b) CONSORT: Any changes to trial outcomes after the trial commenced, with reasons**		
"All participants (N = 1,256) were recruited from a large public tertiary hospital in Hanoi, Vietnam."		
**7a) CONSORT: How sample size was determined**		
**7a-i) Describe whether and how expected attrition was taken into account when calculating the sample size**		
**7b) CONSORT: When applicable, explanation of any interim analyses and stopping guidelines**		
"The scores of work engagement in both intervention groups improved from baseline to 3-month follow-up but slightly decreased by the 7-month follow-up. The score in the control group kept increasing from baseline to 7-months."		
**8a) CONSORT: Method used to generate the random allocation sequence**		
"A stratified permuted block random table was generated by an independent biostatistician."		
**8b) CONSORT: Type of randomisation; details of any restriction (such as blocking and block size)**		
Stratified permuted-block randomization was conducted. The block sizes were fixed at 3. Participants were stratified according to the baseline depression subscale score of DASS 21 into two strata (≥10 or <10) [42].		
**9) CONSORT: Mechanism used to implement the random allocation sequence (such as sequentially numbered containers), describing any steps taken to conceal the sequence until interventions were assigned**		
Enrollment was conducted by a CRC (TTTT), and the assignment was conducted by an independent research assistant. The stratified permuted-block random table was password protected and blinded to the researchers. Only the research assistant could access it during random allocation.		
**10) CONSORT: Who generated the random allocation sequence, who enrolled participants, and who assigned participants to interventions**		
Stratified permuted-block randomization was conducted. The block sizes were fixed at 3. Participants were stratified according to the baseline depression subscale score of DASS 21 into two strata (≥10 or <10) [42]. A stratified permuted block random table was generated by an independent biostatistician. Enrollment was conducted by a CRC (TTTT), and the assignment was conducted by an independent research assistant. The stratified permuted-block random table was password protected and blinded to the researchers. Only the research assistant could access it during random allocation.		
**11a) CONSORT: Blinding - If done, who was blinded after assignment to interventions (for example, participants, care providers, those assessing outcomes) and how**		
**11a-i) Specify who was blinded, and who wasn’t**		
N/A		
**11a-ii) Discuss e.g., whether participants knew which intervention was the “intervention of interest” and which one was the “comparator”**		
**11b) CONSORT: If relevant, description of the similarity of interventions**		
N/A		
**12a) CONSORT: Statistical methods used to compare groups for primary and secondary outcomes**		
For the main pooled analysis, a mixed model for repeated measures conditional growth model analysis with an unstructured covariance matrix was conducted, using a group (intervention and control) × time (baseline, 3-month, and 7-month follow-ups) interaction as an indicator of the intervention effect. The two intervention effects (Program A vs Control and Program B vs Control) were simultaneously tested in the model. For sensitivity analysis, a similar mixed model for repeated measures, but using the analysis of variance model, with an unstructured covariance matrix, was conducted.		
**12a-i) Imputation techniques to deal with attrition / missing values**		
At the baseline survey, if the number of missing items was less than half of the number of total items, the missing values were imputed, using values calculated according to the following equation: (the mean value x the number of total items) / the number of missing items.		
**12b) CONSORT: Methods for additional analyses, such as subgroup analyses and adjusted analyses**		
For sensitivity analysis, a similar mixed model for repeated measures, but using the analysis of variance model, with an unstructured covariance matrix, was conducted.		
RESULTS		
**13a) CONSORT: For each group, the numbers of participants who were randomly assigned, received intended treatment, and were analysed for the primary outcome**		
The participant flowchart is shown in Figure 1. In total, 75.8% of workers (962/1,269) participated in the baseline survey (September 2018). After 11 were excluded, 951 met the eligibility criteria. Finally, the 951 participants were randomly allocated with 317 in each group (two interventions and one control group).		
**13b) CONSORT: For each group, losses and exclusions after randomisation, together with reasons**		
The participant flowchart is shown in Figure 1.		
**13b-i) Attrition diagram**		
**14a) CONSORT: Dates defining the periods of recruitment and follow-up**		
In total, 75.8% of workers (962/1,269) participated in the baseline survey (September 2018).		
**14a-i) Indicate if critical “secular events” fell into the study period**		
**14b) CONSORT: Why the trial ended or was stopped (early)**		
N/A		
**15) CONSORT: A table showing baseline demographic and clinical characteristics for each group**		
The baseline characteristics of participants are shown in Table 1.		
**15-i) Report demographics associated with digital divide issues**		
The baseline characteristics of participants are shown in Table 1.		
**16a) CONSORT: For each group, number of participants (denominator) included in each analysis and whether the analysis was by original assigned groups**		
**16-i) Report multiple “denominators” and provide definitions**		
"A process evaluation showed similar completion rates for both programs (83.3% for Program A and 86.1% for Program B) [33]. " in Discussion.		
**16-ii) Primary analysis should be intent-to-treat**		
**17a) CONSORT: For each primary and secondary outcome, results for each group, and the estimated effect size and its precision (such as 95% confidence interval)**		
N/A		

**17a-i) Presentation of process outcomes such as metrics of use and intensity of use**		
**17b) CONSORT: For binary outcomes, presentation of both absolute and relative effect sizes is recommended**		
Program B showed a significant estimated effect for improving work engagement at 3 months (t = 1.97, p = 0.049, d = 0.16 [95%CI: 0.001 - 0.43]).		
**18) CONSORT: Results of any other analyses performed, including subgroup analyses and adjusted analyses, distinguishing pre-specified from exploratory**		
N/A		
**18-i) Subgroup analysis of comparing only users**		
**19) CONSORT: All important harms or unintended effects in each group**		
N/A		
**19-i) Include privacy breaches, technical problems**		
**19-ii) Include qualitative feedback from participants or observations from staff/researchers**		
DISCUSSION		
**20) CONSORT: Trial limitations, addressing sources of potential bias, imprecision, multiplicity of analyses**		
**20-i) Typical limitations in ehealth trials**		
Limitation<br>The study has several limitations. First, participants were recruited from a single hospital in Vietnam and were limited to full-time nurses with a personal smartphone. The hospital is a large and prestigious general tertiary hospital in Hanoi. Therefore, the generalizability of these findings to the wider nursing population is limited. Second, this study did not adopt a stratified randomized method by using work engagement scores. Therefore, we did not conduct a subgroup analysis. Further studies on improving work engagement by stratified randomization might achieve a larger effect size in the low work engagement population. Third, all outcomes in this study were measured by self-report, which might be affected by participants' perceptions or institutional factors. Future studies should consider the use of additional objective outcome measures, such as supervisor ratings of work performance. Fourth, the possibility of contamination of information for the control group, which may have reduced differences between intervention and control groups, resulting in possible underestimation of intervention effectiveness, was not fully controlled in this study. Fifth, besides the intervention programs, an informal group chat (via Viber, Zalo, Facebook Messenger) led by researchers and hospital head nurses that was used to increase the participation rate may also have contributed to improvement of work engagement in the intervention and control groups. Sixth, on the other hand, there may have been social pressure or frustrations caused by frequent reminding to study the programs [33], which could reduce the effect of the program on work engagement. Seventh, the programs were designed mainly to target smartphone users, while they can be used via a PC or tablet. Nurses who did not have smartphones or Internet access were excluded from access to the programs. In a future trial, in addition to the smartphone-based program, the same content of the programs should also be provided via other delivery modes, such as computer or tablet or a booklet. Finally, it is not known whether a similar effect of this program would be observed outside of the nursing profession, in Vietnam or in other LMICs. Future studies should explore the generalizability of the present findings to occupations other than nurses and in other LMICs.		
**21) CONSORT: Generalisability (external validity, applicability) of the trial findings**		
**21-i) Generalizability to other populations**		
**21-ii) Discuss if there were elements in the RCT that would be different in a routine application setting**		
**22) CONSORT: Interpretation consistent with results, balancing benefits and harms, and considering other relevant evidence**		
**22-i) Restate study questions and summarize the answers suggested by the data, starting with primary outcomes and process outcomes (use)**		
The three-arm RCT examined the effects of a newly developed smartphone-based stress management program to improve work engagement as the secondary outcome at 3- and 7- month follow-ups among hospital nurses in Vietnam. Program B, which was a six-module CBT program with fixed sequential order, showed a significant small intervention effect on work engagement at the 3-month follow-up (d=0.16, p=0.049).		
**22-ii) Highlight unanswered new questions, suggest future research**		
Other information		
**23) CONSORT: Registration number and name of trial registry**		
Trial Registration: UMIN Clinical Trials Registry (UMIN-CTR) UMIN000033139. (https://upload.umin.ac.jp/cgi-open-bin/ctr/ctr_view.cgi?recptno=R000037796)		
**24) CONSORT: Where the full trial protocol can be accessed, if available**		
Imamura, K., et al., Effects of two types of smartphone-based stress management programmes on depressive and anxiety symptoms among hospital nurses in Vietnam: a protocol for three-arm randomised controlled trial. BMJ Open, 2019. 9(4): p. e025138.		
**25) CONSORT: Sources of funding and other support (such as supply of drugs), role of funders**		
This research was supported by the Japan Agency for Medical Research and Development (AMED) under Grant Number JP17jk0110014 and JP18jk0110014. The funder had no role in study design, data collection, data analysis, decision to publish or preparation of the manuscript.		
**X26-i) Comment on ethics committee approval**		
**x26-ii) Outline informed consent procedures**		
**X26-iii) Safety and security procedures**		
**X27-i) State the relation of the study team towards the system being evaluated**		
